# Efficacy, immunogenicity and safety of HPV vaccination in Chinese population: A meta-analysis

**DOI:** 10.3389/fpubh.2023.1128717

**Published:** 2023-02-17

**Authors:** Jianming Guo, Shuyan Guo, Siping Dong

**Affiliations:** ^1^National Institute of Hospital Administration, National Health Commission, Beijing, China; ^2^Southern Institute of Pharmacoeconomics and Health Technology Assessment, Jinan University, Guangzhou, China; ^3^School of Political Science and Public Administration, Wuhan University, Wuhan, China; ^4^Health Research Institute, Fujian Medical University, Fujian, China

**Keywords:** HPV vaccine, meta-analysis, efficacy, immunogenicity, safety

## Abstract

**Objective:**

To evaluate the efficacy, immunogenicity and safety of HPV vaccination in Chinese population.

**Methods:**

PubMed, Embase, Web of Science and Cochrane Library from inception to November 2022 were searched to collect information on clinical trials of HPV vaccines. Database search strategy used a combination of subject terms and free terms. Studies were first identified by two authors through reading the title, abstract and full texts and, subsequently, based on the inclusion criteria: Chinese population, with at least one of the following outcomes (efficacy, immunogenicity, and safety), and HPV vaccine RCT, those eligible were included in this paper. Efficacy, immunogenicity and safety data, pooled by random effects models, are presented as risk ratios [95% confidence intervals (CI)].

**Results:**

Eleven RCTs and four follow-up studies were included. Meta-analysis showed that HPV vaccine had good profile of efficacy and immunogenicity. The seroconversion rates were significantly higher among the vaccinated, uninfected (initial negative serum antibody) population than the placebo population for both HPV-16 (RR 29.10; 95% CI: 8.40–100.82) and HPV-18 (RR 24.15; 95% CI: 3.82–152.84), respectively. A significant reduction of the incidence of cervical intraepithelial neoplasia grade 1 (CIN1+) (RR 0.05; 95% CI: 0.01–0.23) and CIN2+ (RR 0.09; 95% CI: 0.02–0.40) was also measured. Risk for serious adverse events after HPV vaccination indicated comparable outcomes between vaccination and placebo.

**Conclusions:**

For Chinese populations, HPV vaccines enhance the level of HPV16- and HPV18-specific antibodies and reduce the incidence of CIN1+ and CIN2+ in uninfected population. Also, the risk of serious adverse events in both groups are almost equivalent. More data are needed to establish vaccine efficacy with cervical cancer.

## 1. Introduction

Cervical cancer is a preventable disease, which can be curable if detected early and treated adequately (1). Yet it is the fourth most common cancer among women and the fourth leading cause of cancer death. In 2020, there were 604,000 new cases of cervical cancer and 342,000 deaths worldwide, an increase in both new cases and deaths compared with that of 2018. Whereas in China, such number of new cases and deaths were 109,740 and 59,060 respectively. China is among the countries with the greatest disease burden of cervical cancer, which calls for prompt and effective measures to eliminate cervical cancer (2, 3).

As is well known that 99.7% of cervical cancers are caused by HPV (4), while HPV 16 and 18 are known to cause at least 70% of cervical cancers (5). Moreover, certain HPV type infection can also lead to anal cancer, vulvar cancer, penile cancer, oral cancer and head and neck cancer (6). A meta-analysis (7) estimated, amongst women with normal cytological findings, the global prevalence of infection with any HPV genotype to be 11.7%; China has a higher prevalence (15.6%) (8). Prevalence of HPV infection were generally associated with HIV infection and men who have sex with men, the former with a higher prevalence.

Evidence from clinical trials (9–12) supports HPV vaccination and clearly demonstrates that different types of HPV vaccines induce high levels of antibodies, prevent HPV vaccine type-related infection and reduce the number of people developing cervical intraepithelial neoplasia grade 1 or higher (CIN1+) and persistent infection (PI). Furthermore, most clinical trials concluded that HPV vaccine is generally safe and well tolerated, with no significant difference in adverse events (AEs) and serious adverse events (SAEs) between the vaccinated and control groups. In addition, vaccination with the 2-valent HPV vaccine and the 4-valent HPV vaccine can provide cross-protective efficacy against some 9-vaccine type HPVs (HPV types 31, 33, and 45) (13).

The 2020 WHO's Global Strategy to Accelerate the Elimination of Cervical Cancer as a Public Health Problem (1) provides a roadmap, through three key targets: vaccination, screening and treatment. This global call-to-action is aiming to reach one goal: to accelerate the elimination of cervical cancer worldwide. To achieving this vision by 2030, China and other 193 countries support this strategy and make efforts to get 90% of girls fully vaccinated with the HPV vaccine by age 15.

Previous studies with population-level evidence of HPV vaccine were generally from developed countries, and few has focused on the Chinese context. Vaccine efficacy, immunogenicity and safety may be affected by the demographical factors, such as race, individual behavior and lifestyle (14). Therefore, cautions should be taken when interpreting these studies from developed countries. In such context, a targeted review on HPV vaccine efficacy, immunogenicity and safety data in the setting of China will be of importance for future introduction of HPV vaccine into national immunization programs.

In this study, we performed a review on clinical trials and follow-up studies on HPV vaccination in China and then conduct a meta-analysis of those studies.

## 2. Methods

### 2.1. Inclusion/exclusion criteria

The inclusion criteria used in this paper focused on the following considerations: (1) Population: Chinese women and/or men of aged 9–45 years were included; (2) Intervention: 2-valent, 4-valent and 9-valent HPV vaccination were selected; (3) Control: vaccination with placebo, or 2-valent, 4-valent and 9-valent HPV vaccination were selected; (4) Outcome: efficacy indicators such as incidence of CIN1+ or CIN2+, immunogenicity indicators such as antibody seroconversion rate were measured and safety indicators such as incidence of local AEs; (5) Study type: randomized clinical trials (RCTs) and follow-up study for RCTs were assessed.

The exclusion criteria adopted were non-English studies; incomplete articles such as conference abstracts; multiple publications of the same randomized clinical trial (only the latest one included); phase I clinical trials and duplicate studies.

### 2.2. Search strategy

We searched PubMed, Embase, Web of Science and Cochrane Library databases for RCTs of HPV vaccines from the time of database establishment to November 2022. The search was conducted using a combination of subject terms and free terms, the detailed PubMed search strategy is (Vaccines, Papillomavirus [Title/Abstract] OR Papillomavirus Vaccine [Title/Abstract]OR Vaccine, Papillomavirus [Title/Abstract] OR Human Papillomavirus Vaccines [Title/Abstract] OR Papillomavirus Vaccines, Human [Title/Abstract] OR Vaccines, Human Papillomavirus [Title/Abstract] OR HPV Vaccine [Title/Abstract] OR Vaccine, HPV [Title/Abstract] OR Human Papilloma Virus Vaccine [Title/Abstract] OR HPV Vaccines [Title/Abstract] OR Human Papilloma Virus Vaccines [Title/Abstract] OR Human Papillomavirus Vaccine [Title/Abstract] OR Papillomavirus Vaccine, Human [Title/Abstract] OR Vaccine, Human Papillomavirus [Title/Abstract] OR Papillomavirus Vaccines [Mesh]). AND (randomized controlled trial [pt] OR controlled clinical trial [pt] OR randomized [tiab] OR placebo [tiab] OR drug therapy [sh] OR randomly [tiab] OR trial [tiab] OR groups [tiab] NOT (animals [mh] NOT humans [mh]) AND (China OR Chinese). Details of additional database search strategy see Supplementary Figure 1. In addition, ClinicalTrials.gov were searched to supplement the trial data and a manual search of references for the included articles was performed.

### 2.3. Data extraction and outcome assessments

All RCTs conducted in China, reporting at least one of the following indicators: efficacy, immunogenicity and safety were included in this paper. Data were extracted independently by two reviewers using a predefined data extraction form and then cross-checked by these two reviewers as well. Disagreements, if any, were resolved through discussion and consultation with a third reviewer. Based on the Preferred Reporting Items for Systematic Reviews and Meta-Analyses (PRISMA) statement (15), the following data were extracted: authors, year published, study types, protocol number, study outcome, participants, vaccine studied, intervention, comparator, outcomes of efficacy, outcomes of immunogenicity, outcomes of safety and funding source.

To perform the meta-analysis, efficacy (CIN1+, CIN2+, 6-month PI and incident infection), immunogenicity (seroconversion rate and Geometric Mean Titer) and safety (local, systemic and serious AEs) data from included articles were extracted. The efficacy indicators were measured by HPV DNA testing throughout the study; the immunogenicity indicators were measured 1 month after the last vaccination by enzyme-linked immunosorbent assay (2-valent) and competitive Luminex immunoassay (4-valent and 9-valent); for the safety indicators, local AEs were reported 3–7 days after each vaccination; systemic AEs were reported 30 days after each vaccination; and serious AEs were reported throughout the study.

For efficacy, CIN is the term for precancerous lesion affecting the cells on the surface of the cervix. CIN1+ is defined as CIN grades 1, 2, and3, low-grade cervical glandular intraepithelial neoplasia (LCGIN), high grade cervical glandular intraepithelial neoplasia (HCGIN), adenocarcinoma in-situ (AIS) or invasive cervical cancer. CIN2+ is defined as CIN grades 2 and 3, LCGIN, HCGIN, AIS or invasive cervical cancer. 6-month PI is defined as having a persistent cervical infection for a specified HPV type if there was a sequence of positive HPV DNA samples for that HPV type, not interrupted by negative samples, such that the total range was more than 5 months (>150 days) apart. Incident infection is defined as at least one positive specified HPV type DNA PCR assay at the time point considered.

For immunogenicity, seroconversion is defined as HPV specific antibody concentration above the cut-off point, and the seroconversion rate is the proportion of participants being seropositive. Immune response to a vaccine is often measured by Geometric Mean Titer (GMT), rise in titer indicating a better vaccine immunogenicity.

For safety, local AEs have three indicators: pain, redness and swelling; systemic AEs under evaluation were fatigue, fever, headache and myalgia. SAEs assessed include medical occurrences that result in death, are life-threatening, require hospitalization or prolongation of hospitalization, result in disability/incapacity or are a congenital anomaly/birth defect in the offspring of a study subject.

### 2.4. Risk of bias assessment and data analysis

The Cochrane Collaboration (16) used in this paper offers a specific tool for assessing risk of bias in each included RCT and the assessing criteria is defined as “low risk,” as “high risk,” or as “unclear risk.”

We focused on efficacy, immunogenicity, and safety data from the rest of the included studies and conducted a meta-analysis of such data. Meta-analysis was performed using Review Manager Software (RevMan version 5.3) for Windows. Relative risks (RR) were calculated in the vaccinated groups and control groups using a random effects model with 95% confidence intervals (CI). Subsequently, heterogeneity between studies was addressed using Cochrane Q test results and quantifying the I^2^ score, where I^2^ value ranges from 0% to 100%, and if it is greater than or equal to 50%, a high degree of heterogeneity is considered. Lastly, Results with high heterogeneity were analyzed in order to find factors that might influence the study results.

## 3. Results

### 3.1. Article selection process

**Figure 1:**
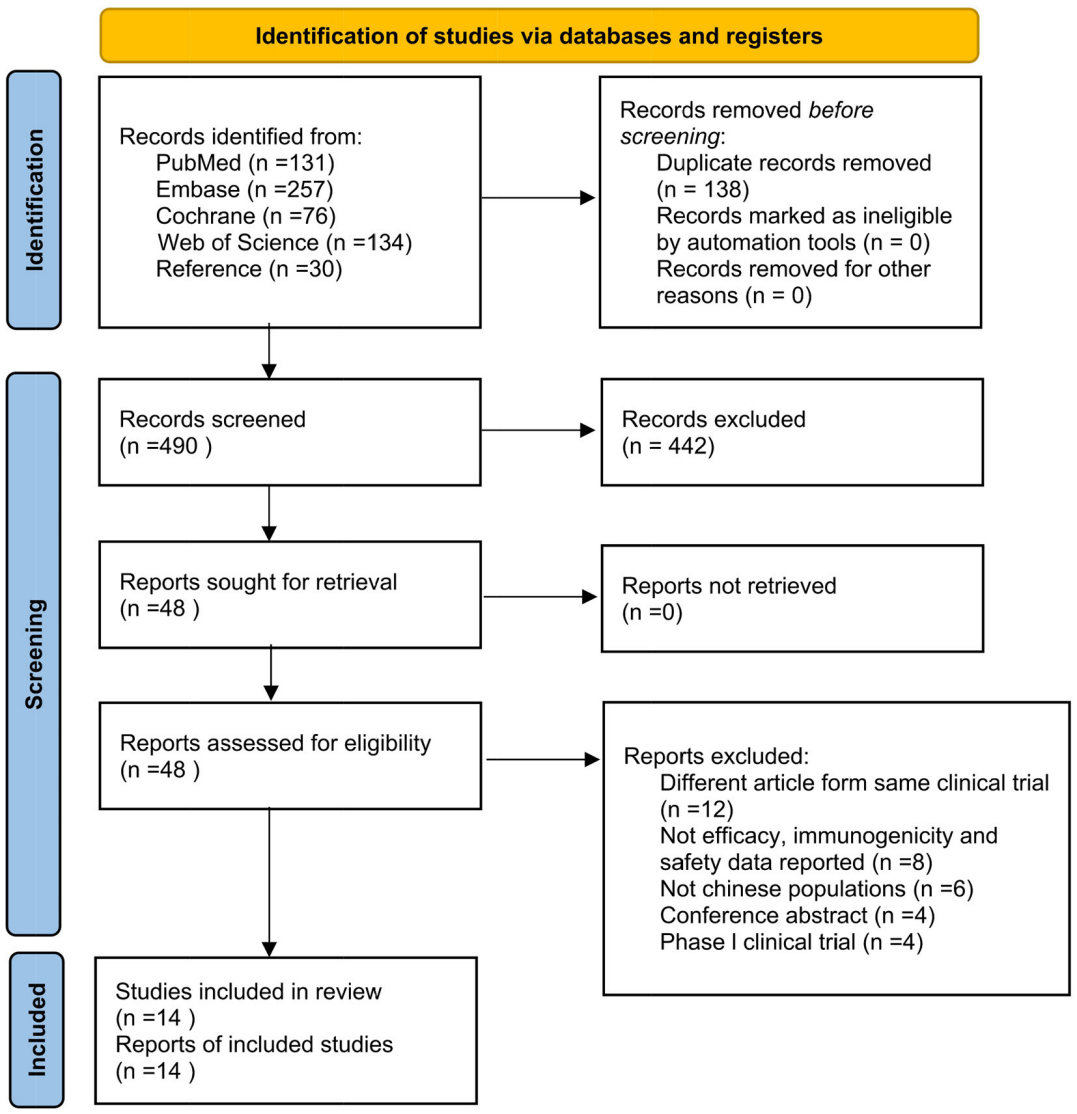
Flow chart of article screening and selection process.

A total of 628 articles were obtained after the initial search, where two researchers independently reviewed the articles, browsed the titles and abstracts, and then read the full text. If any disagreement appeared, a third reviewer was consulted. Eventually, 14 studies were included (17–30), containing 11 RCTs (17–26) and four follow-up studies (27–30). The specific article screening process is shown in Figure 1.

### 3.2. Characteristics of the included studies

The basic characteristics of the included studies were shown in Table 1. All included RCTs were randomized double-blind controlled trials except one (19) randomized observer-blinded controlled trial; more than half (*n* = 6) (17–20, 26) were single-center trials, the rest being multi-center trials; All 11 (17–26) RCTs were with registration numbers, where ten (17, 19–26) out of the 11 RCTs were with women subjects only, age ranging from 9 to 45 years.

**Table 1:** Basic characteristics of studies included in the review.

**Study**	**Ngan et al. (17)**	**Li et al. (18) and Huang et al. (27)**	**Zhu et al. (19)**	**Zhu et al. (19)**	**Wu et al. (20)**	**Chen et al. (21), Wei et al. (24), and Zhao et al. (28)**	**Garland et al. (25)**	**Zhu et al. (22) and Zhao et al. (29)**	**Qiao et al. (23) and Zhao et al. (30)**	**Shu et al. (26)**
Study tapes	RDSCT	RDSCT	RDSCT	ROSCT	RDSCT	RDMCT	RDMCT	RDMCT	RDMCT	RDSCT
Vaccine studied	2-valent vs. placebo	4-valent vs. placebo	2-valent vs. placebo	2-valent vs. placebo	2-valent vs. placebo	4-valent vs. placebo	9-valent vs. 4-valent	2-valent vs. placebo	2-valent vs. placebo	9-valent vs. 4-valent vs. 4-valent
Protocol number	NCT00306241	NCT00496626	NCT00996125	NCT01277042	NCT01356823	NCT00834106	NCT00543543; NCT00943722	NCT00779766	NCT01735006	NCT04425291
Assessed outcomes	Immunogenicity and safety	Immunogenicity and safety	Immunogenicity and safety	Immunogenicity and safety	Immunogenicity and safety	Efficacy and safety	Efficacy, immunogenicity and safety	Efficacy, immunogenicity and safety	Efficacy, immunogenicity and safety	Immunogenicity and safety
Participants	Women aged 18–35 years	Males aged 9–15 years and Females aged 9–45 years	Girls aged 9–17 years	Women aged 26–45 years	Women aged 18–25 years	Women aged 20–45 years	Women aged 9–26 years	Women aged 18–25 years	Women aged 18–45 years	Women aged 20–45 years
Intervention group	150 vaccinated with 2-valent HPV vaccine	302 vaccinated with 4-valent HPV vaccine	374 vaccinated with 2-valent HPV vaccine	606 vaccinated with 2-valent HPV vaccine	400 vaccinated with 2-valent HPV vaccine	1,503 vaccinated with 4-valent HPV vaccine	345 vaccinated with 9-valent HPV vaccine	3,689 vaccinated with 2-valent HPV vaccine	3,026 vaccinated with 2-valent HPV vaccine	1,120 vaccinated with 4-valent or 9-valent HPV vaccine
Control group	150 vaccinated with placebo	298 vaccinated with placebo	376 vaccinated with placebo	606 vaccinated with placebo	400 vaccinated with placebo	1,503 vaccinated with placebo	346 vaccinated with 4-valent HPV vaccine	3,683 vaccinated with placebo	3,025 vaccinated with placebo	560 vaccinated with 4-valent HPV vaccine
Outcomes of immunogenicity	Seroconversion rates and GMTs	Seroconversion rates and GMTs	Seroconversion rates and GMTs	Seroconversion rates and GMTs	Seroconversion rates and GMTs	/	Seroconversion rates and GMTs	Seroconversion rates and GMTs	Seroconversion rates and GMTs	Seroconversion rates and GMTs
Outcomes of efficacy	/	/	/	/	/	CIN1+, CIN2+, PI, incident infection	Incident infection	CIN1+, CIN2+, PI, incident infection	CIN1+, CIN2+, PI, incident infection	/
Outcomes of safety	AEs, SAEs	AEs, SAEs	AEs, SAEs	AEs, SAEs	AEs, SAEs	AEs, SAEs	AEs, SAEs	AEs, SAEs	AEs, SAEs	AEs, SAEs
Funding source	GSK	Merck	GSK	GSK	National Natural Science Foundation of China	Merck	Merck	GSK	National Natural Science Foundation of China	National Natural Science Foundation of China

RDSCT, randomized, double-blind, single-center, controlled trial; ROSCT, randomized, observer-blind, single-center, controlled trial; RDMCT, randomized, double-blind, multi-center, controlled trial; HPV, human papilolomavirus; GMT, Geometric Mean Titer; GSK, GlaxoSmithKline; CIN, cervical intraepithelial neoplasia; PI, persistent infection; AEs, adverse events; SAEs, serious adverse events; /, not reported.

With regards to the vaccine types, subjects were vaccinated with 2-valent HPV vaccine (GSK) (14, 16, 19), domestic 2-valent HPV vaccine (the National Natural Science Foundation of China) (17, 20), domestic 4-valent HPV vaccine and domestic 9-valent HPV vaccine (the National Natural Science Foundation of China) (23), 4-valent HPV vaccine (Merck) (15, 18), and 9-valent HPV vaccine (Merck) (22). All the control groups were on a placebo except 9-valent HPV vaccine trials. For vaccination procedures, RCTs with 2-valent HPV vaccine were to vaccinate at Months 0, 1 and 6, while RCTs with 4-valent HPV vaccine and 9-valent HPV vaccine were at Months 0, 2 and 6.

Seroconversion occurs when the antibody concentration value is above the cut-off point. For 2-valent HPV vaccine (GSK), the value was 8 EU/ml for HPV-16 and 7 EU/ml for HPV-18; for domestic 2-valent HPV vaccine, one trial (20, 23) adopted an increase in antibody titers of at least fourfold and the other (23) used 3.1 IU/ml for HPV-16 antibodies and 2.0 IU/ml for HPV-18; for 4-valent HPV vaccine and 9-valent HPV vaccine, the value was 20 mMU/ml (milli-Merck unit/ml) and 24 mMU/ml as cut-off points for antibodies against HPV16 and HPV18.

For clinical outcomes assessed, five RCTs (21–23, 25) reported HPV vaccine efficacy; ten RCTs (17–20, 22, 23, 25, 26) reported immunogenicity of HPV vaccine; all 11 RCTs (17–26) reported the safety of the HPV vaccine. The efficacy, immunogenicity and safety data were collected from the total vaccinated set or per-protocol set.

Four follow-up studies (27–30) reported long-term immunogenicity and safety with follow-ups up to 3.5 years, 66 mouths, 8 years and 10 years. As follow-up study has a variable follow-up timeline and the control group of 9-valent HPV vaccine RCTs was not on a placebo, therefore a descriptive analysis on 9-valent HPV vaccine RCTs and follow-up study was conducted.

### 3.3. The risk of bias assessment

**Figure 2:**
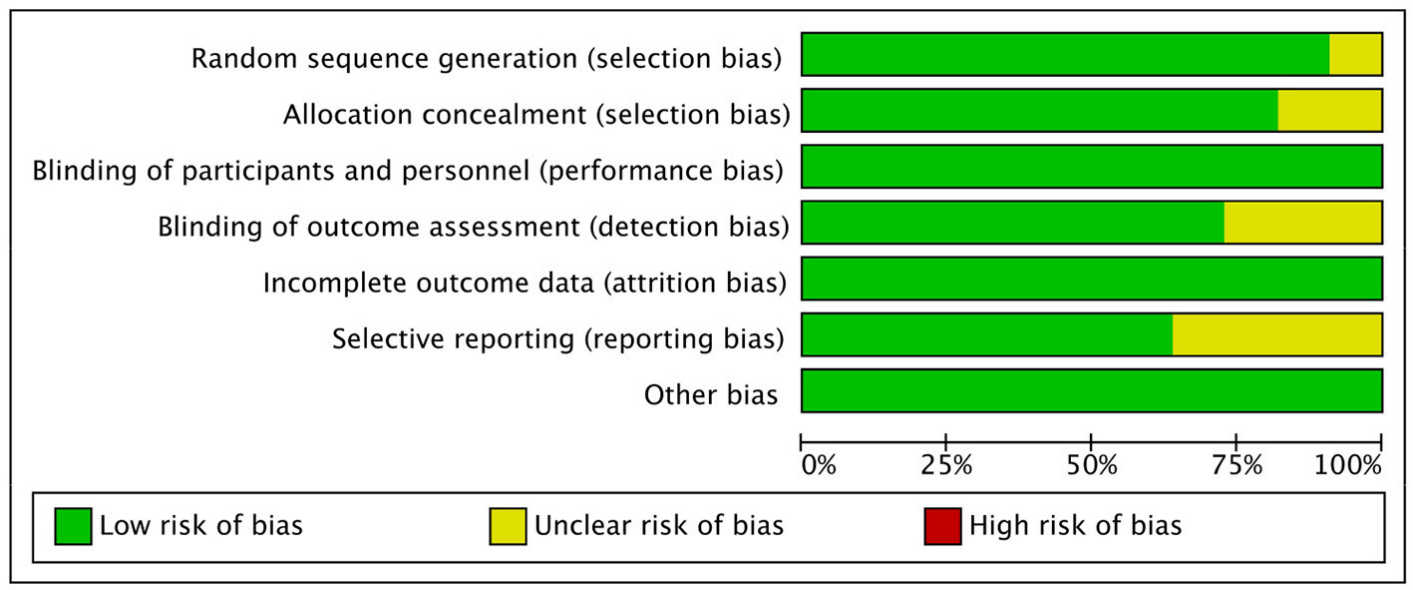
Risk of bias.

In terms of selection bias, all trials (*n* = 11) were randomized trials, with only ten trials (17, 19–26) describing the method of sequence generation, and nine (17, 19, 20, 22–26) detailing the unpredictability of random allocation of subjects. In order to maintain low risk of bias, all trials were blinded and unlikely to break; for bias in measurement of the outcomes, eight trials (19, 21–26) had explicit blindness for outcomes measurement and were unlikely to break. All 11 trials had complete results and no missing outcome data, with four (19, 22, 23) reporting bias, due to the lack of data for the control groups (Figure 2).

### 3.4. Immunogenicity of HPV vaccine

**Figure 3:**
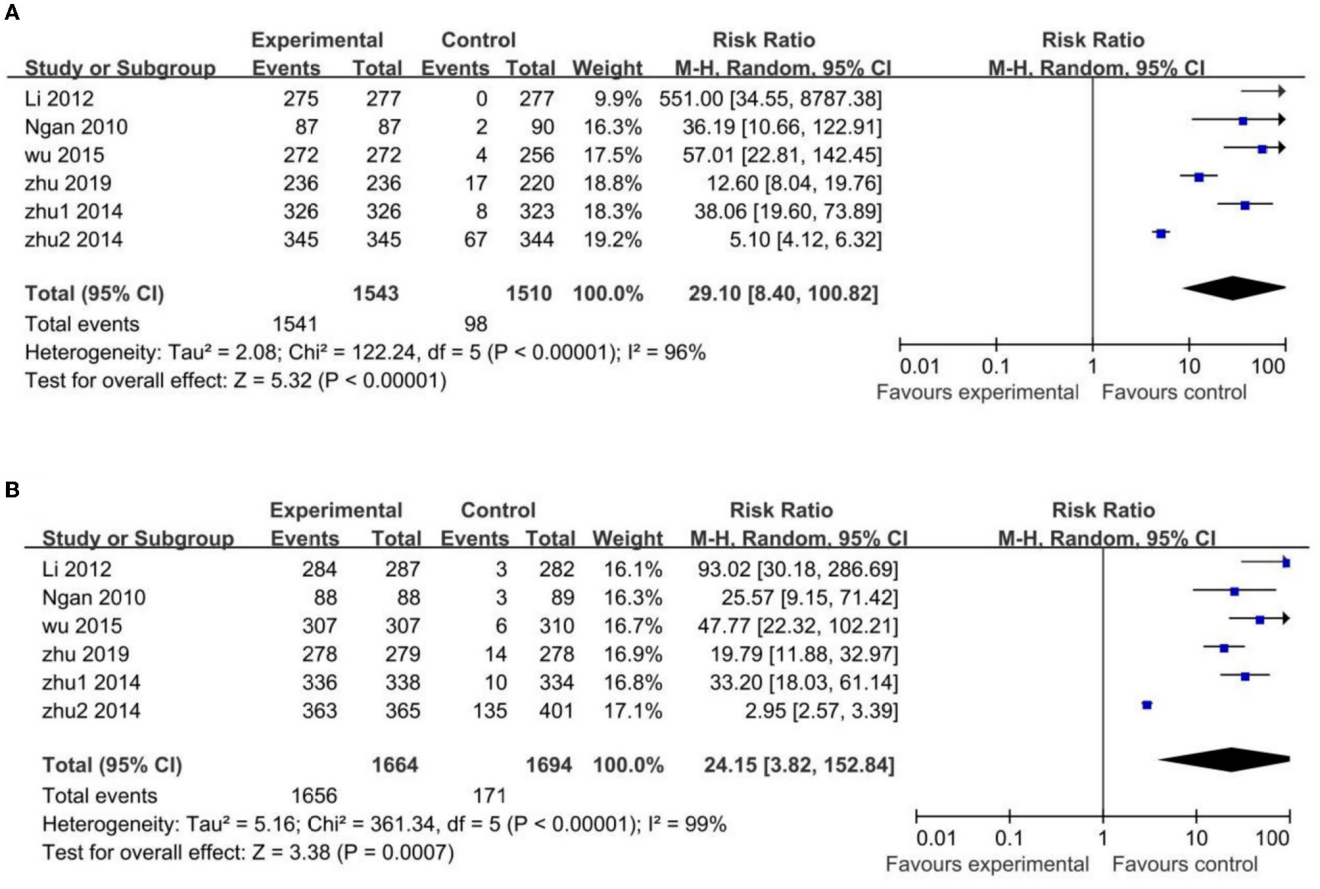
Comparison HPV vaccines vs. control in HPV-16 **(A)** and HPV-18 **(B)** antibody seroconversion rate in uninfected Chinese population.

Based on the published clinical data, the HPV-16/18 antibody seroconversion rates at Month 7 after three doses of HPV vaccine in the initial antibody serum negative (uninfected) population were calculated. And pooled RR of seroconversion rate between vaccinated groups and control groups was analyzed with a random-effects model (Figure 3).

The uninfected population's HPV-16 antibody seroconversion rate was higher in the vaccinated groups than those in the control groups, with a statistically significant difference (RR 29.10; 95% CI: 8.40–100.82). However, the heterogeneity among the pooled studies was high (*I*^2^ = 96%), due to two trials (19, 22) with narrow confidence intervals and disproportionate difference in their mean estimates. In particular, one (19) of these two trials with high heterogeneity showed huge significance with the other trials (RR 5.10; 95% CI: 4.12–6.32). In the sensitivity analysis excluding these two trials, the difference between the vaccinated and control groups remained statistically significant (RR 49.63; 95% CI: 24.93–98.80), but less heterogeneous (I^2^ = 38%) (Supplementary Figure 2).

Similar results occurred in the HPV-18 antibodies: the uninfected population's HPV-18 antibody seroconversion rate was higher in the vaccinated groups than that in the control groups, with a statistically significant difference (RR 24.15; 95% CI: 3.82–152.84). High heterogeneity was also found in these pooled studies (I^2^ = 99%), with the same two trials (19, 22) of heterogeneity for same reason, and the same one trial (19) of greatest heterogeneity (RR 2.95; 95% CI: 2.57–3.39). In the sensitivity analysis excluding these two trials the difference between the vaccinated and control groups remained statistically significant (RR 40.93; 95% CI: 25.96–64.53) but with reduced heterogeneity (I^2^ = 17%) (Supplementary Figure 3).

In addition, GMT of HPV-18 and HPV-16 in the uninfected populations were highly heterogeneous and the high heterogeneity could not be reduced. We did not perform a meta-analysis on such data, but for individual trials, GMT and seroconversion rates were significantly and statistically higher in the vaccinated groups than those in the control groups, for uninfected populations.

### 3.5. Efficacy of HPV vaccine

Three HPV vaccine trials were included in meta-analysis of efficacy, two (22, 23) for 2-valent HPV vaccine and one (21) for 4-valent HPV vaccine. Only one (22) out of the three clinical trials reported efficacy data at Months 24, 48, 57 and 72, the rest two with only data at Months 42 and 78.

**Figure 4:**
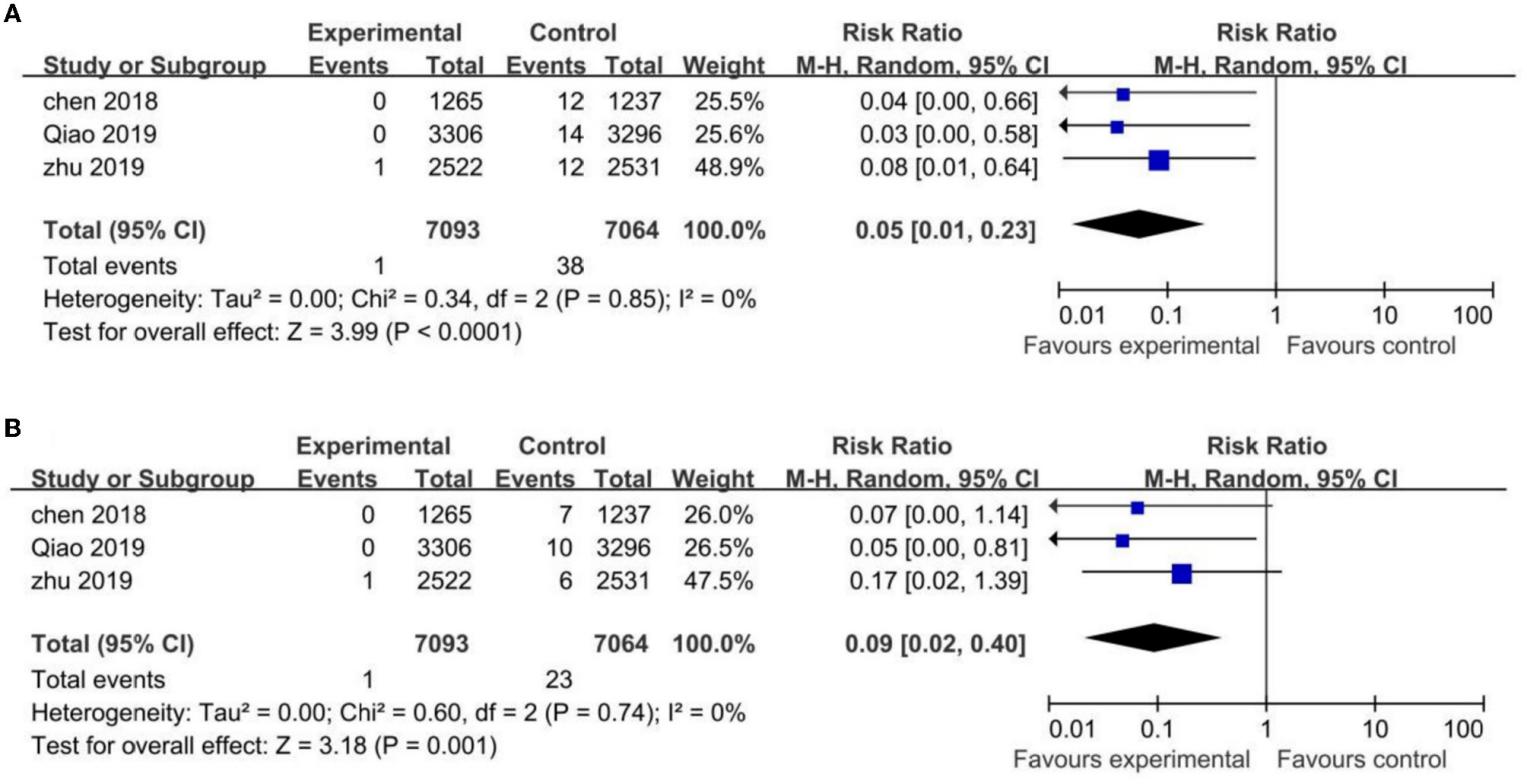
The risk of CIN1+ **(A)** and CIN2+ **(B)** with HPV vaccination vs. control.

The three trials mentioned above showed statistically significant differences in the incidence rate of CIN1+ and CIN2+ associated with HPV-16/18 in the vaccinated groups compared with the control groups, with (RR 0.05; 95% CI: 0.01–0.23, I^2^ = 0) for CIN1+ and (RR 0.09; 95% CI: 0.02–0.40, I^2^ = 0) for CIN2+ (Figure 4).

Two out of the three trials (22, 23) evaluated the 6-month PI and incident infections, and for the uninfected population, such data was statistically lower in the vaccinated groups than those in the control groups. The RR values were (RR 0.04; 95% CI: 0.01–0.12, I^2^ = 0) for the 6-month PI and (RR 0.28; 95% CI: 0.21–0.36, I^2^ = 0) for incident infections (Supplementary Figures 4, 5).

### 3.6. Safety of HPV vaccine

**Figure 5:**
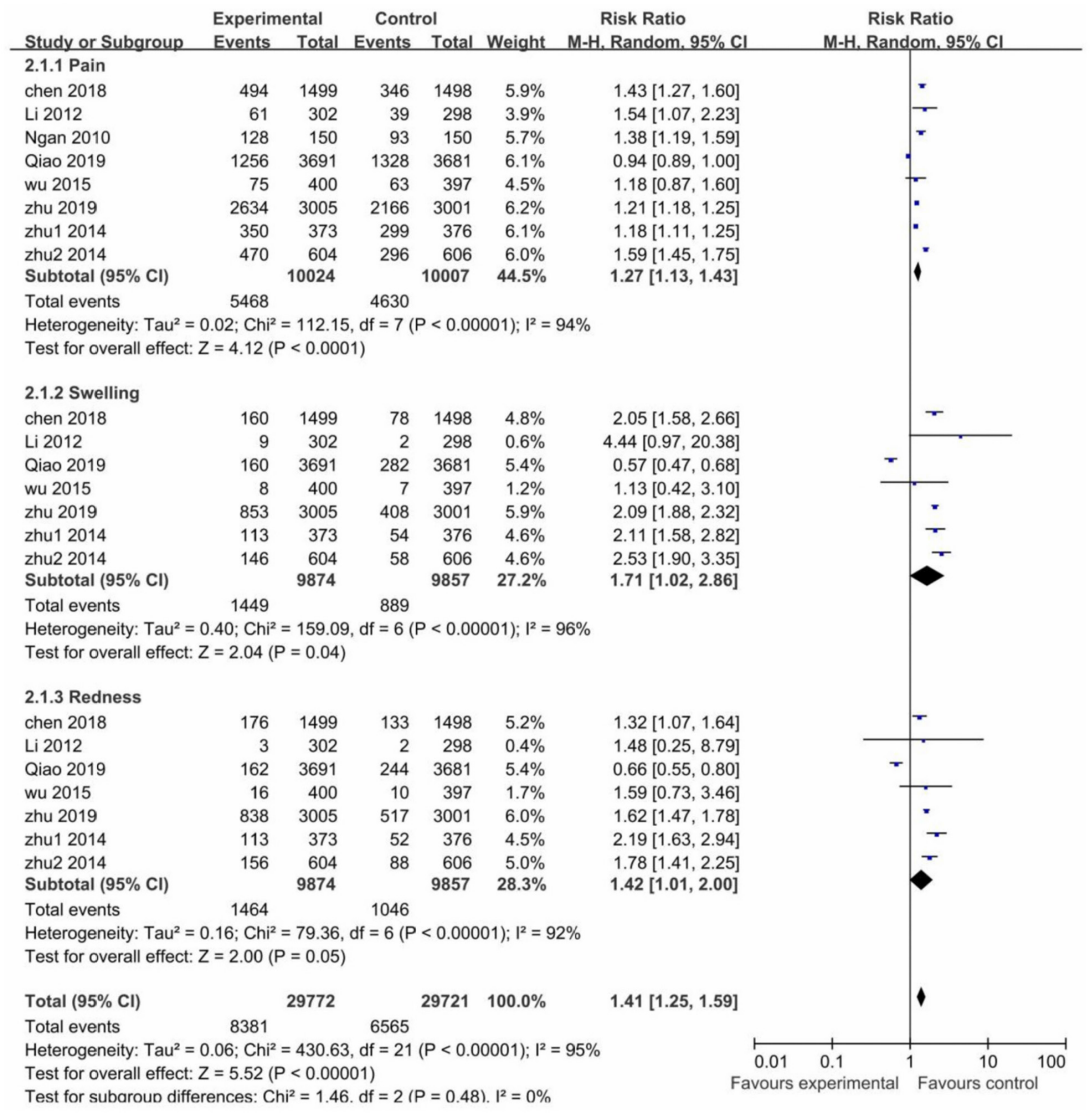
The risk of local adverse events.

In most of the trials, local AEs were reported within 3–7 days after each vaccination in both the vaccinated and control groups. The risk of pain, swelling and redness after vaccination was higher in the vaccinated groups than the control groups, RR values were (1.27; 95% CI: 1.13–1.43) for pain, (1.71; 95% CI: 1.02–2.86) for swelling, (1.42; 95% CI: 1.01–2.00) for redness. However, all pooled studies were presented with high heterogeneity (I^2^ > 75%). Nevertheless, the risk of local adverse reactions was higher in the vaccinated groups than in the control groups (RR 1.41; 95% CI: 1.25–1.59) (Figure 5).

**Figure 6:**
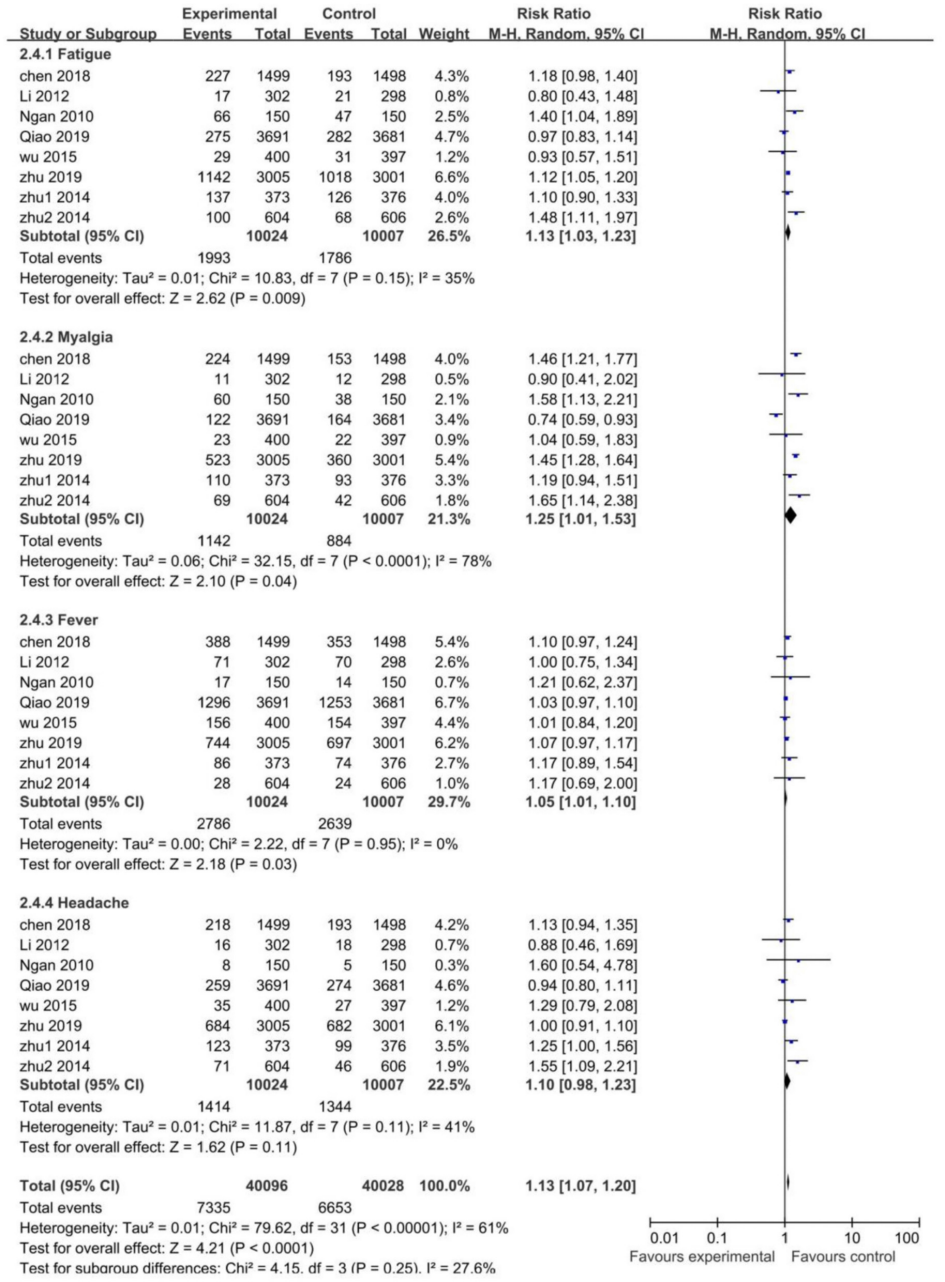
The risk of systemic adverse events.

Systemic AEs occurred within 30 days after each vaccination in the studies. The risk of fatigue (RR 1.13; 95% CI: 1.03–1.23), myalgia (RR 1.25; 95% CI: 1.01–1.53), and fever (RR 1.05; 95% CI: 1.01–1.10) were higher in the vaccinated groups. The risk of headache was similar between the vaccinated and control groups (RR 1.10; 95% CI: 0.98–1.23). For the heterogeneity, it was acceptable in all pooled studies (I^2^ <50%), except for myalgia with high heterogeneity (I^2^ = 78%). Conclusively, the risk of systemic AEs was higher in the vaccinated groups than the control groups (RR 1.13; 95% CI: 1.07–1.20) (Figure 6).

Through sensitivity analysis, it is found that Qiao's study (23) contributed to the high heterogeneity. A domestic 2-valent HPV vaccine was used in Qiao's study, and we assumed that the type of vaccine may be the main reason for heterogeneity in local and systemic AEs, which led to a subgroup analysis of vaccine types. For all trials, two (20, 23) evaluated the domestic 2-valent HPV vaccine, whose subgroup analysis showed that the risk of both local and systemic AEs in the vaccinated groups was similar to that in the control groups and only pain (I^2^ = 51%) and redness (I^2^ = 78%) with heterogeneity. After removing the domestic 2-valent HPV vaccine trials, the non-domestic 2-valent HPV vaccine subgroup reported a decreased heterogeneity in most local and systemic AEs, with swelling (I^2^ = 96% → I^2^ = 0%) and myalgia (I^2^ = 78% → I^2^ = 0%) decreased to no heterogeneity and redness (I^2^ = 92% → I^2^ = 51%) to moderately high heterogeneity (Supplementary Figures 6–9).

All trials reported SAEs throughout the study. As study duration varied, a period of 7–12 months was selected for SAEs assessment. Data from six trials (17–20, 23) showed that the risk of SAEs after HPV vaccination in the vaccinated groups was similar to the control groups (RR at 1.04; 95% CI: 0.64–1.71, I^2^ = 0%) (Supplementary Figure 10).

### 3.7. Follow-up study

Four HPV vaccine RCTs with follow-ups were included in this study and the HPV vaccines used in the follow-up studies were imported 2-valent (26), domestic 2-valent (27) and imported 4-valent (24, 25). The follow-up period for these four RCTs were 10 years, 66 months, 3.5 years and 8 years, respectively.

One study (27) reported the long-term immunogenicity and the data showed that IgG antibody positivity for HPV-6,−8,−16 and−18 remained above 80% in the female population 3.5 years after the first vaccination. In the male population, all HPV-6,−8,−16 and−18 IgG antibodies remained above 90%. Three studies (28–30) reported the long-term efficacy of HPV vaccination, with a strong prevention of HPV-related precancerous diseases during the follow-up period, and over 90% efficacy on HPV16/18 CIN1+. Two studies (29, 30) showed long-term safety and HPV vaccination is safe and well tolerated, with no vaccine-related SAEs reported.

### 3.8. Nine-valent HPV vaccines

Two 9-valent HPV vaccine RCTs were included, including one imported 9-valent HPV vaccine trial (25) and one domestic 9-valent HPV vaccine trial (26). The imported 9-valent HPV vaccine global multi-centers RCT reported data from Hong Kong and Taiwan sub-centers, which investigated the efficacy, immunogenicity and safety of imported 9-valent HPV vaccines in two age groups (9–15 years, 16–26 years). The results showed that the seroconversion rates of each vaccinated group were >98%, no persistent infection lasting more than 6 months, and no vaccine-related SAEs reported. While the domestic 9-valent HPV vaccine trial was a non-inferiority trial compared with imported 4-valent HPV vaccines, it evaluated the immunogenicity and safety of domestic 9-valent HPV vaccines, domestic 4-valent HPV vaccines and imported 4-valent HPV vaccines in three age groups (20–26 years, 27–35 years, and 36–45 years). The results showed that the seroconversion rates of each group were >99%, and the incidence of AEs was comparable among these three groups.

## 4. Discussion

A search on ClinicalTrials.gov for HPV vaccines showed that most of the HPV vaccine clinical trials conducted to date have been in developed countries mostly in European countries, the United States and Japan (31–33). With good clinical practices and adequate financial support, such clinical trial results help to speed up the process of HPV vaccine into the immunization programs in developed countries. For developing countries, a more severe disease burden calls for an urgent need of domestic clinical trial data on HPV vaccines. Based on the previous experience on rotavirus vaccine (34), it is aware that the clinical trials results from developed countries cannot be simply applied to the developing countries. Demographic variables such as race would result in different levels of vaccination immunogenicity and vaccine efficacy between developed and developing countries. Following the call from the WHO's strategy for cervical cancer elimination, a growing need to conduct the state-specific clinical trials of HPV vaccines in particular among Chinese population can be foreseen.

The results of the meta-analysis showed that HPV vaccination in the uninfected population produced high vaccine efficacy and strong prevention of PI and CIN1+ in most cases, which resembles previous studies (35) in other regions. However, because the natural course of cervical cancer expands up to dozens of years, it is difficult to observe cervical cancer during the follow-up timeline set in the current studies. Further follow-up studies are suggested to fill the research gap.

This meta-analysis showed that HPV vaccination in the Chinese uninfected population produced a high degree of immunogenicity, as evidenced by high seroconversion rate in vaccinated groups, similar to previous studies and meta-analysis (36, 37) published in markets other than China. It is found that comparing only one indicator—seroconversion rates between the vaccinated and control groups is incomplete to reach a solid conclusion on vaccine immunogenicity because the GMT of seropositive subjects in the control groups was much lower than those in the vaccinated groups. Nevertheless, studies suggested that HPV vaccine has the potential to bring long-term immune efficacy.

The meta-analysis revealed vaccine immunogenicity with high heterogeneity. For HPV-16 and HPV-18 antibody seroconversion rate in the uninfected population, the sensitivity analysis found that two trials (19, 22) caused heterogeneity. This heterogeneity was remarkably induced by one trial (19). The vaccinated groups in both trials (16, 19) had comparable results with other trials, but the presence of more seropositive subjects in the control groups makes heterogeneity inevitable. Upon comparison, it is noted that subjects age ranged between 26 and 45 years in Zhu's study (16), and those in other trials ranged between 9 and 35 years, suggesting age seems to correlate with increased seropositive subjects. Although the other study (22) included Chinese women aged 18–25, due to its exclusion criteria—“women with no sexual experience due to culture and ethics were excluded,” subjects in this study may have more sexual activities than those in other studies. As a national study (38) from China stated that the median age at sexual debut was 22 years in urban China and 21 years in rural China. More sexual activity may contribute to an increase in the number of seropositive controls. Studies suggested that older age and virginity loss are the cause of heterogeneity. In fact, both factors lead to more frequent sex in the study population, which in turn increases the probability of becoming seropositive through natural infection in the control groups.

With regard to HPV vaccine safety, our meta-analysis showed that the Chinese population is tolerant to AEs from the HPV vaccines, and the risk difference of AEs for both vaccinated and control groups were low. Local AEs and some systemic AEs (fatigue, myalgia, fever), however, were more common in the vaccinated groups and with high heterogeneity. When exploring the reasons behind heterogeneity, a subgroup analysis of HPV vaccine types was performed. The analysis revealed that the risk of local and systemic AEs was similar between the vaccinated and control groups for the Chinese 2-valent HPV vaccine, and a remarkably lower heterogeneity was seen upon exclusion of both trials. This suggests that the Chinese 2-valent HPV vaccine may have a better safety profile in the Chinese population. Moreover, AEs of other HPV vaccines were also tolerable, which was consistent with previous studies (39, 40). Last but not the least, there were no significant differences between the vaccinated and control groups in terms of SAEs and vaccine-related SAEs.

There are several limitations in this study: first, a subgroup analysis of the 11 trials was impossible because the age intervals in these trials overlapped, and only one trial included Chinese women aged 26–45 years, which could not provide sufficient data to determine the age effect on the clinical outcomes of HPV vaccination; second, the clinical trials of 4-valent and 9-valent HPV vaccines in the Chinese population were lacking. Two clinical trials of 4-valent HPV vaccine were included in our study, but one missing immunogenicity data, one missing efficacy data, and the control groups in three 9-valent HPV vaccine trials included were not on placebo and lacking efficacy data, therefore it was not possible to perform a subgroup analysis of the vaccine's valence type; third, data on the outcome of HPV vaccination in Chinese men were limited with only one clinical trial of the 4-valent HPV vaccine included 100 male subjects. Fortunately, when searching ClinicalTrials.gov for ongoing clinical trials in China, several 4- and 9-valent HPV vaccine clinical trials are in progress, which could further supplement the data on HPV vaccine in the Chinese population in the future.

In the WHO position paper (41), the primary target population for HPV vaccination recommended is girls aged 9–14 years prior to their first sexual intercourse, and a vaccine catch-up program is recommended to be initiated for women aged ≥15 years. This important milestone provides China with an additional option for future HPV vaccine clinical trials: the study population of the trials could be further stratified by age subgroup. In this context, the heterogeneity among clinical trials can be reduced. Furthermore, age stratification for HPV vaccination may also help to present the real-world data once it enters the national immunization program.

Although China has promoted cervical cancer screening to control the increase in cervical cancer cases, China still accounts for 1/6 of the world's cervical cancer new cases and deaths cases in 2020 due to its large population base. What is worse, HPV vaccine, a primary strategy for preventing cervical cancer, is a self-pay vaccine in China. The high price and lack of knowledge on HPV vaccine determine low coverage of HPV vaccine in China. The inclusion of the vaccine in the national immunization program requires a comprehensive evaluation, including a cost-benefit analysis and a budget impact analysis, besides the safety and efficacy evaluation. There is still a long way to go for HPV vaccine inclusion in the national immunization program, but in the face of the current severe burden of cervical cancer, we are eager to see that happen as soon as possible.

## 5. Conclusion

For Chinese populations, HPV vaccines enhance the level of HPV16- and HPV18-specific antibodies and reduce the incidence of CIN1+ and CIN2+ in uninfected population. Also, the risk of serious AE in both groups are almost equivalent. More data are needed to establish vaccine efficacy with cervical cancer.

## Data availability statement

Publicly available datasets were analyzed in this study. This data can be found here: PubMed, Embase, Web of Science, and Cochrane Library databases.

## Author contributions

JG performed the material preparation, data analysis, and the first draft of the manuscript. SG contributed to data collection, the conception of the study, modification opinions, and was responsible for polishing the article. SD contributed significantly to analysis and manuscript preparation, helped perform the analysis with constructive discussions, and polish the article to make it more readable. All authors commented on previous versions of the manuscript, read, and approved the final manuscript.
